# Shape change in the saddle region of the equine back during trot and walk

**DOI:** 10.1098/rsif.2023.0644

**Published:** 2024-06-19

**Authors:** Kristina P. Smirnova, Michael A. Frill, Sharon E. Warner, Jorn A. Cheney

**Affiliations:** ^1^ Royal Veterinary College, Hatfield AL9 7TA, UK; ^2^ School of Biological Sciences, University of Southampton, Southampton SO17 1BJ, UK

**Keywords:** saddles, horse, photogrammetry, back shape

## Abstract

Equine back pain is prevalent among ridden horses and is often attributed to poor saddle fit. An alternative explanation is that saddle fits are technically good but fit to the wrong configuration. Saddles are fit for the standing horse, but much of the time ridden is instead spent locomoting when the back experiences the greatest peak forces. We used an array of cameras to reconstruct the surface of the back and its movement during trot, walk and standing for five horses. We verified the setup’s accuracy by reconstructing a laser-scanned life-sized model horse. Our reconstructions demonstrate that saddles sit within a large, relatively low-mobile region of the back. However, saddles do sit adjacent to the highly mobile withers, which demands care in positioning and design around this important region. Critically, we identified that saddle curvature between standing and moving horses is substantially different, where trotting and walking horses have flatter backs than their standing configurations. Saddles designed around the locomoting configuration of horses may improve horse welfare by being better fit and decreasing the focal pressures applied by saddles.

## 1. Introduction

Equine back pain is prevalent among ridden horses and is often attributed to poor saddle fit. Conservative estimates suggest back pain is seen in at least 35% of ridden horses [1–4]. Furthermore, significant back traumas, often attributed to incorrectly fitted saddles, constitute between 8% and 10% of equine musculoskeletal injuries [5–7] as poor saddle fit leads to abnormally high focal pressure on regional tissues [8–10].

Saddles are designed and assessed with a stationary horse [11]; however, of all the tissues of the back, only the osseous components of the equine thoracolumbar spinal column are particularly immobile [12,13]. The rigidity of the equine thoracolumbar spinal column is owing to the tightly spaced articulations, relative thinness of the discs between the vertebral bodies and the numerous intervertebral ligaments [14–16]. These adaptations restrict the lateral deviations of the spinal column and allow a limited degree of dorsoventral flexion–extension, which likely contributes to the efficient locomotion of horses [12,14].

The epaxial spinal muscles of horses activate regionally during locomotion [13,17]. Their gait-dependent activity changes along the length of the back and is asymmetrical (about the sagittal plane) in both activity and intensity [17–19]. These temporally and spatially distinct patterns signify that, despite a relatively rigid skeletal frame, the overlaying soft tissue supporting the saddle is dynamic [20]. Certainly, saddle placement around the extrinsic forelimb muscles of the withers (an elevated region containing the cranial thoracic and caudal cervical vertebrae along with the dorsal border of the scapulae) can negatively affect locomotion, seemingly resulting from saddle interference with muscular and scapular mobility [9,21–24].

A major cause of epaxial muscle injury is improperly fitted saddles [25]. Saddle-induced compression results in muscle atrophy, which manifests in shortened stride length, increased spinal column stiffness and decreased locomotor performance of horses [26], in addition to pain and behavioural changes [10,27]. Beyond atrophy, 26% of horses with suspected back injuries have epaxial muscle damage [28] likely owing to high saddle-induced pressures [10,23,29,30]. Therefore, it is essential to better distribute the pressure of the saddle and rider over the soft tissues of the back.

Here, our aims were to measure the regional morphing of the equine back during locomotion to understand how soft-tissue movement could lead to high pressures and tissue damage and to determine whether this could be accomplished using photogrammetry with high-speed cameras. We measured shape change across the equine back in trot and walk, using photogrammetric high-speed surface reconstructions to create a map of the most- and least-mobile areas of the equine back for the two gaits. This map may be critical for future saddle design to indicate the areas of the back most appropriate for distributing pressure and, thus, the regions best justified for supporting the compressive forces of the saddle and rider.

## 2. Methods

### 2.1. Horses

Five horses were recruited for this study from volunteers in the Greater London area. Horses were between 8 and 17 years of age, with body-condition scores of either three or four on a scale of five (where one is unhealthily thin and five is unhealthily obese).

The study population was five leisure horses and included two Irish sports horses, two Connemaras and one pony. Coat lengths were comparable among the horses. All horses exercised regularly but were of varied fitness conditions (table 1). Horses were led by their owners and were provided with water and treat rewards.

**Table 1.** Details of horses examined in this study.

horse ID	breed	age (years)	significant history/exercise	height at withers (m)	weight (kg)	body-condition score	lameness	post-flexion testing
1	Irish sport horse	8	regular competitions	1.7	536	3/5	sound	sound
2	Connemara	8	transient lamenesses resolved. Saddle problems in past. No longer used for riding	1.5	468	4/5	sound	sound
3	sports pony	11	previously competed, now low-level regular exercise	1.52	477	4/5	sound	sound
4	Connemara	17	exercises four times weekly. Mild lameness diagnosed at study and owner informed	1.51	485	4/5	1/10 RF	1/10 RF
5	Irish sport horse	13	regular exercise	1.46	522	4/5	sound	sound

RF, right forelimb.

Before entry into the study, each horse underwent a two-stage vetting (including validating documentation, a full clinical history, a full clinical examination and lameness assessment at walk, trot and post-flexion testing). None of the horses were undergoing veterinary treatment (other than routine preventative care), and all were identified as sound by their owners. Only one individual was identified as 1/10 lame on one forelimb during our veterinary exam. The owners of all horses were present during the study, informed of the examination results and gave permission for data collection following the two-stage vetting. The study’s veterinary surgeon (member of the Royal College of Veterinary Surgeons, no. 7330790) was present throughout to continually assess the gait and to promote good welfare and practice.

The technique that we used for surface reconstruction required additional colour to be added to the hair coat to provide a better visual texture. The monochromatic colouring of many horses can hinder surface reconstruction, as the method relies on identifying visual features on the horses seen by multiple cameras. To improve the reconstructions, we distributed chalk powder (The Hatchwell Company Limited, Rishton UK) and/or activated charcoal (granular charcoal animal feed supplement; The Dorset Charcoal Company Ltd, Hazelbury Bryan, UK) onto the fur to increase the number of detectable features and their accuracy; lighter coats received charcoal, darker coats received chalk and neutral-coloured coats received both. After the study, the chalk and charcoal were gently brushed off the coats. We selected chalk and activated charcoal as they are regarded as gut-safe for horses if ingested [31,32].

### 2.2. Experimental overview

**Figure 1.**
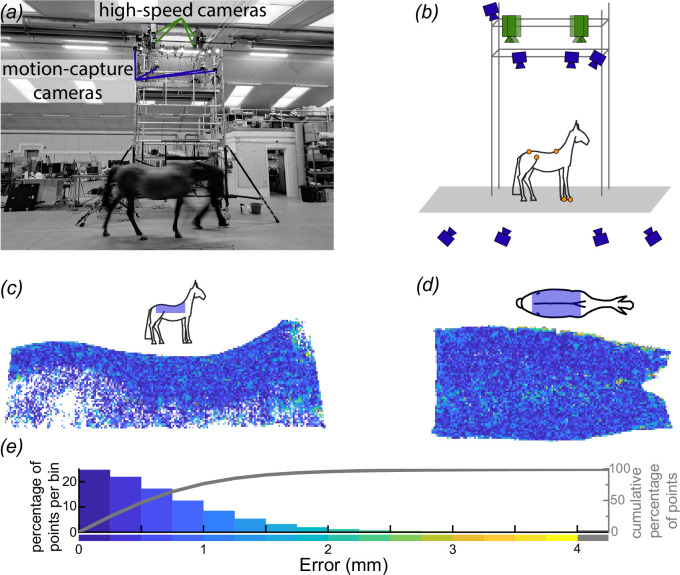
Schematic of our equine back-surface-measurement apparatus and evaluation of its accuracy. (*a*,*b*) The study used two arrays of cameras: four high-speed (green) and eight motion-capture cameras (blue) for measuring back surface, as well as gross kinematics and stride timing, respectively. We positioned motion-capture cameras both above the horse on the tower to identify the orientation and position of the saddle region and level with the horse to identify timings of forelimb contact with the ground; both sets of parameters were derived using motion-capture markers (orange circles in *b*). (*c–e*) We estimated reconstruction accuracy by reconstructing a life-size, rigid, horse model and compared the surface generated by our high-speed-camera array to that of a professional laser scan of the rigid model. We present the differences in the surface between the laser-scanned model and our measurement apparatus in right lateral (*c*) and dorsal (*d*) views and summarized (*e*) as a histogram and plot of the cumulative percentage of surface points contained across error thresholds (grey line, right *y*-axis). (*c*,*d*) Insets present schematic views of the rigid horse model and the approximate location of the reconstructed surface in blue.

Dynamic surface measurements of the equine back were conducted indoors in an elongated open-plan research ‘barn’ at the Structure & Motion Laboratory of the Royal Veterinary College (Hatfield, UK). Prior to the start of each trial, horses were allowed to acclimate to the research space. During each trial, a horse was guided by its handler along a path parallel to a rigid mobile access tower with overhanging cameras (figure 1*a*). The area below the cameras was the measurement volume, and the handler was asked to maintain a constant speed through this 2 m long volume (see electronic supplementary material, table S1, for summary statistics of the speed). Horses were allowed to establish their preferred speed. The handler and horse had 15 m to establish the gait, trot or walk, and accelerate to speed prior to entering the volume. After exiting the measurement volume, the handler and horse had 20 m to decelerate comfortably, typically requiring only 12 m.

We imaged the dorsal surface of a horse’s back using four synchronized, high-speed cameras (FASTCAM SA3; Photron Europe Limited, West Wycombe, UK) mounted at the top of the rigid tower (figure 1*a,b*). Illumination for the images was provided by custom stroboscopic light emitting diode lamps (comprised of Bridgelux Vero 29; Digi-key Electronics, Thief River Falls, USA). Imaging was synchronized with a motion-capture system (Oqus 300 & 500 series; Qualisys AB, Göthenburg, Sweden), which estimated stride timings and location of the horse within the measurement volume. We tracked six retroreflective markers in total: two on the hooves, two on the pelvis and two on the spine. Markers on the dorsal aspect of the hoof of each forelimb provided timings of contact with the ground and related change in back surface geometry to instantaneous timing within the stride cycle. Markers on the tuber coxae (cranial-most point of the iliac wings of the pelvis) provided anatomical reference. Markers along the spinal column, a cranial marker over the dorsal spinous process of vertebra T6 and one at the midpoint between the greater trochanter of each femur (at the level of caudal vertebra 1, Cd1) were used to locate the orientation and position of the equine back within the measurement volume (figure 1*b*). Locating the horse in the volume allowed efficient cropping or masking away of extraneous surfaces not of interest, for example, the floor or the handler. We placed additional markers, not used in this study, on the hooves of the hindlimbs and the poll.

We collected data for eight trot and five walk trials through the measurement volume for each horse. We expected each walk trial to contain at least one full stride within the measurement volume; however, in trot, depending upon the speed, trials might not capture a full stride. We estimated that if we captured 60% more trots than walks, we would record at least a sample size of five for any instant in the stride, even with relatively long stride lengths.

### 2.3. Decentring lenses relative to sensors to capture parallel image planes

Reconstructing the surface of flying birds and bats had been previously done using similar camera configurations to those in this study (e.g. [33,34]). Here, we developed this photogrammetric technique further by better aligning the image planes through lateral shifting of the lenses.

**Figure 2.**
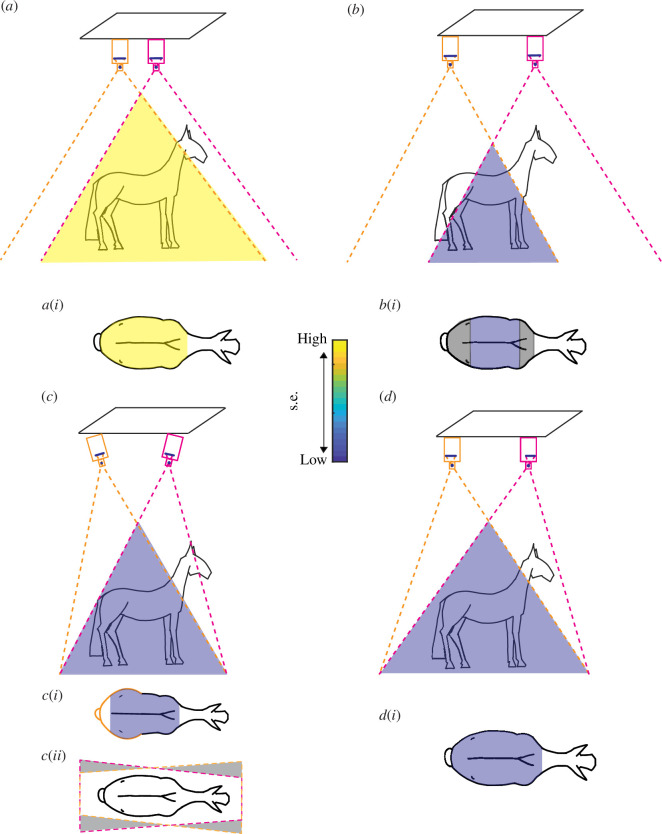
Schematics of theoretical paired-camera configurations and associated images of the horizontal back surface. (*a*) Close-together vertically oriented cameras result in large overlap in fields of view but (*a*,i) low accuracy. (*b*) Further-spaced vertically oriented cameras result in reduced overlap in fields of view causing (*b*,i) an accurate but small reconstruction volume. (*c*) Tilted further-spaced cameras increase overlap in the fields of view (*c*,i) is more accurate than (*a*) but perspective distortion in the image planes produces non-uniform spatial resolution and leads to (*c*,ii) trapezoidal non-overlapping fields of view with wasted (grey shading) spatial resolution. (*d*) Radial shifting of the lenses (indicated by black circles) relative to the sensors (thick black lines) results in better-aligned fields of view without the need for significant camera tilt and (*d*,i) produces a more accurate reconstruction owing to uniform higher spatial resolution. (*a*,*d*) Scale bar indicates s.e. of the reconstruction.

Typical camera configurations waste substantial portions of the field of view, reducing the spatial resolution of the reconstruction. The most common configurations tilt the cameras towards each other to produce overlapping images that cover a common field of view, i.e. the measurement volume (figure 2*c*). However, tilting the cameras produces perspective distortion, where any rectangular region of interest not parallel to the sensor will appear to contract as it moves further from the sensor owing to the expansion of the actual field of view (figure 2*c*). Additionally, perspective distortion decreases spatial resolution non-uniformly, especially in the regions where fewer pixels define the area of interest. An alternative common configuration is to align the cameras in parallel (figure 2*a,b*). For cameras placed far apart, views barely overlap and most of the image is wasted (figure 2*b*(i)), but if alternatively, cameras are placed close together, while views will overlap, the configuration is less accurate in the out-of-plane direction owing to the views being too similar (figure 2*a*(i)) [35].

Our approach to maximizing spatial resolution was to orient the cameras to be nearly parallel but shift the centre of the lens relative to the centre of the sensor, such that the sensor’s field of view was not straight ahead but instead at an angle (figure 2*d*). By keeping the sensors roughly parallel, it removed perspective distortion between camera pairs. That is, any rectangular region of interest seen by one camera will appear rectangular in another camera, and for any distance orthogonal to the parallel array of cameras, camera placement can achieve identical fields of view (figure 2*d*).

The limitation for how much a lens can be shifted relative to the sensor is determined by the size of the image circle relative to the sensor. We used a lens that produces a large 64 mm diameter circle (PC-E NIKKOR 24 mm f/3.5D ED, Nikon UK), while the diagonal of the cameras’ sensors was 24 mm, which would allow the lens to shift by as much as 20 mm. Our shift was much less substantial, approximately 5 mm in each camera, which equates to tilting each camera by 12°.

### 2.4. Camera calibration

Surface reconstruction required accurate knowledge of the camera’s intrinsic and extrinsic properties. Intrinsic properties included lens distortion, focal length and lens shift. Extrinsic properties included position and orientation. We calibrated the intrinsic properties for each camera individually. To perform the calibration, we mounted each camera on a tripod and collected an array of images of a 1.75 m × 1.45 m checkerboard that nearly filled the field of view of the cameras at a distance of approximately 2 m from the camera, with each image capturing a different position and orientation of the checkerboard. We calibrated the extrinsic properties for the four cameras as a group once mounted on the tower (figure 1*b*) and oriented for image acquisition. During this calibration, a 1 m × 1 m textured board with corner markers was held and moved through the image volume below the cameras, from which we estimated camera position and orientation based on the position of common features and corner markers in the images across the synchronized cameras.

### 2.5. Estimating calibration accuracy and reconstruction error

To estimate the accuracy of our camera calibration and surface reconstruction, we reconstructed the saddle region of the back for a life-size, rigid horse model and compared the surface generated by our high-speed-camera array to that of a professional laser scan (Surface Scan, London, UK) generated with a 20 µm accurate scanner (KSCAN20; Scantech (Hangzhou) Co. Ltd, Hangzhou, China). While laser scanners are exceptionally accurate for rigid structures, they are not designed to cope with a large dynamic surface moving through an even larger volume. Here, the purpose of the laser scanner was to generate a reference surface of the saddle region for the horse model, which we could compare to our reconstruction of the same rigid model to generate an estimate of the accuracy of our camera configuration.

When imaging the rigid horse model with our high-speed camera array, we applied white chalk powder to the black-painted surface of the model to increase the number and accuracy of features detected by the cameras, as we did for live black-coated horses.

### 2.6. Identifying stride cycle timings using motion-capture cameras

The motion-capture cameras recorded a larger volume than the measurement volume for the saddle-region surface data. With this larger volume, we captured forelimb contact and push-off both before and after the horse entered the surface measurement volume. This allowed us to better identify and link the specific instant in the stride cycle to the surface data.

**Figure 3.**
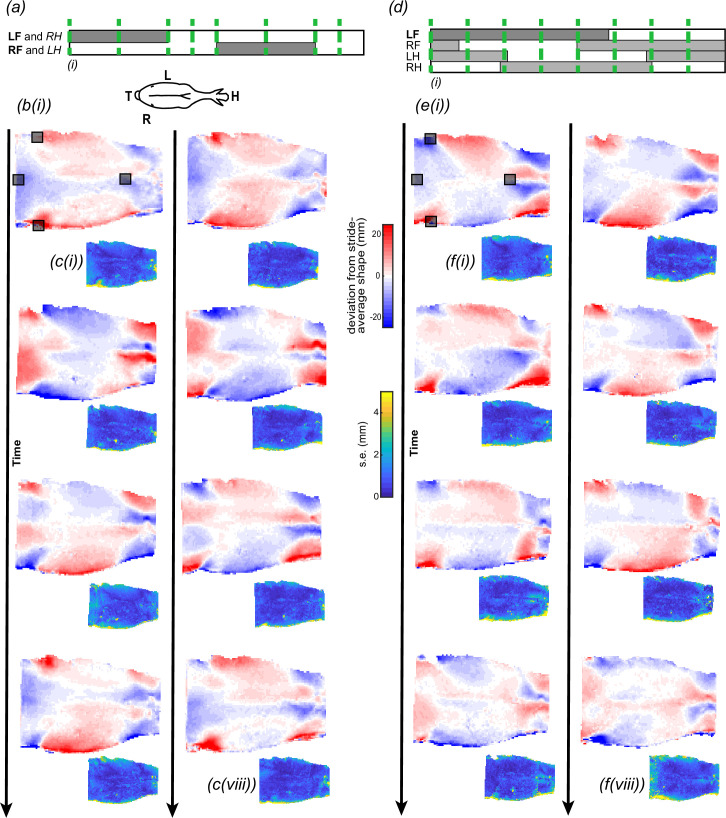
Changes in equine back shape in trot (*a–c*) and walk (*d–f*). (*a*,*d*) We measured back shape (elevation) across eight instances throughout the stride (green dashed lines). Instances of the stride cycle were defined by the footfall patterns of the left and right forelimbs in trot, or the left forelimb in walk. The presentation of hindlimb timings is approximate. (*b*,*e*) Dorsal views of the equine back colour-coded by change in elevation relative to the stride-average shape at eight instances in trot (*b*) and walk (*e*); instances (i–iv) are the first column, and instances (v–viii) are the second column. Comparing across columns within a gait, pairs of stride instances are offset by half of the stride cycle and form approximate mirror images of one another, reflected about the spine. (*c*,*f*) Changes in back elevation were consistent across multiple strides, as demonstrated by the s.e.m., colour-coded across the surface. Typical errors were an order of magnitude less than the peak changes in elevation. The error presented captures errors in identifying the same instances in time, aligning or registering the shapes and measurement errors. Each instant represents the average and s.e. in elevation over (*b*,*c*) 6–8 strides (*n* = (6,7,7,6,7,8,7,8)) at trot, and (*e*,*f*) 5–6 strides (*n* = (5,5,5,6,5,5,5,5)) at walk. (*a*) LF: left forelimb, RH: right hindlimb; RF: right forelimb and LH: left hindlimb. (*a*) Inset of dorsal view of horse displaying H: head, T: tail, L: left, R: right. Grey squares represent motion-capture markers placed at (in cranio-caudal direction): T6, tuber coxae (two markers) and Cd1.

We computed stride cycle timings using the movement of the motion-capture marker(s) for the left forelimb in walk and the left and right forelimbs in trot. We defined periods of hoof contact with the ground (stance phase) as the duration when the motion-capture markers were relatively static, and periods out of contact with the ground (swing phase) when the markers were in motion. Footfall timings were identified by eye. In walk, we selected eight instances evenly spaced in time throughout the stride cycle. In trot, the eight instances were defined kinematically for both the left and right forelimbs to capture the specific instances of forelimb contact with the ground, push-off, midway through contact or midway through the aerial phase (figure 3). We analysed the shape of the back surface for each of the instances in trot and walk. Specific instants in the stride were repeatable and demonstrated little variation in back shape across trials (figure 3*c,f*).

### 2.7. Aligning/registering the surfaces

Each reconstructed surface required cropping of extraneous surface detail, such as the surface of the floor, the handler’s arm or the head or limb of the horse. As the surface was located in different regions of the volume and at different orientations across trials, we placed all of the data within a new coordinate system based around the orientation of the horse: with one axis aligned parallel with the horse’s spinal column, another approximately parallel with gravity and the third orthogonal to the plane formed by those vectors, with the origin placed at the caudal spine marker. This coarse initial alignment allowed us to empirically identify a volume relative to that coordinate system that contained only the region of interest and lacked extraneous features that could affect fine-scale alignment. This coarse alignment and cropping of the surface enabled fine-scale alignment or registration using an ‘iterative-closest-point’ algorithm (pcregistericp.m in Mathworks 2022a; The Mathworks, Inc., Natick, USA), which minimized the disparity among surfaces by accounting for small rotations and translations that might exist in the coarse alignment. If we had not cropped extraneous surfaces, such as the floor, they would have biased and misaligned the surfaces of the horse.

After aligning the surfaces at each instant, we interpolated the ‘vertical’ elevation and depression over a 1 cm × 1 cm grid at each point along all of the surfaces. This was necessary as any surface was described at arbitrary locations and was, in effect, just a dense cloud of points, which would not have allowed for quantitative comparisons across surfaces, such as computing mean vertical movement. Once the surfaces were interpolated onto a common grid, we computed the mean surface elevation at each instant and the standard error of the means (s.e.m.) across the surface. Here, s.e. captured behavioural deviation from the mean, as well as errors in identifying the exact same instances in time, aligning or registering the surfaces and measurement error.

Finally, we described how surface elevation changed over the stride relative to the stride average. For walk, the stride-average surface is the average of eight instances evenly spaced in time. For trot, as the eight instances in time were unevenly spaced, we weighted the contribution of each surface by the duration between temporally adjacent instances in the stride to reconstruct the comparable stride-average surface.

### 2.8. Reconstructions of and comparisons to the standing horse

We recorded the surface of the standing horse’s back twice, before and after the horse underwent a two-stage veterinary examination. Of the two reconstructions, we selected the one most symmetrical. We found no evidence that surface curvature changed systematically between the two reconstructions, which we evaluated by sectioning the standing horse in both reconstructions and comparing surface curvature. Our overall conclusions were not sensitive to which surface we selected; for presentation, we selected the standing shape with the least lateral (left–right) curvature when viewed dorsally (from above). We recorded the standing shape of four horses; no standing data could be collected for horse ID #5.

### 2.9. Deformation mapping

We computed deformation, or shape change, relative to different mean surfaces depending upon the comparison of interest. To describe the instantaneous change in shape over eight instances in the stride, we first estimated the trial-average shape at eight instances in the stride defined by footfall patterns and compared each instance of the trial-average shape to that of the stride-average shape. The stride-average shape presents the minimum ‘chronic’ changes in elevation and depression possible (as opposed to minimizing the acute changes that would reflect the average between peak elevation and peak depression). To describe the difference between the shape of the horse when moving and standing, we compared the stride-average shape in trot or walk to the standing shape.

## 3. Results

While we recorded and analysed the back shape change of five horses (table 1), to simplify figure presentation, we focused primarily on a single horse (horse ID #1). Equivalent figures for the other horses are provided either in the results or the electronic supplementary material. The gross patterns of shape change in the back were consistent among all five horses (electronic supplementary material, figures S1–S6), and our conclusions apply to all five horses in our sample. Our analysis focuses on the dorsoventral displacement of the back tissue relative to either the stride-average or standing shape of the back surface after alignment; we refer to dorsal displacement as elevation and ventral displacement as depression. We describe the single horse using the mean surface of the repeated strides, and we provide summary descriptions of the range of mean surfaces reconstructed for each of the five individuals. As the horses were of different breeds, backgrounds, ages, body-condition scores and three-dimensional back shapes, we did not compute statistics across the five individuals, as this would have made the results more abstract and required morphing the horses to a common shape.

**Figure 4.**
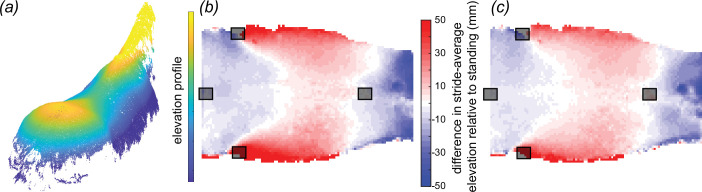
The surface of the standing horse (*a*) has greater curvature longitudinally (concave up) and transversely (concave down) than that of the moving horse at trot (*b*) or walk (*c*). (*a*) Elevation profile of the horse’s standing shape, recorded after veterinary examination. (*b*,*c*) The difference in the stride-average elevation profile relative to the standing horse for trot (*b*) and walk (*c*). The stride-average elevation profiles for both gaits exhibit flatter surfaces, where the depressed lateral regions elevate and the elevated cranial and caudal regions depress. The profile in trot (*b*) is further flattened relative to walk (*c*), which is flatter relative to standing (*a*). Grey squares represent motion-capture markers placed at (in the cranio-caudal direction): T6, tuber coxae (two markers) and Cd1.

**Figure 5.**
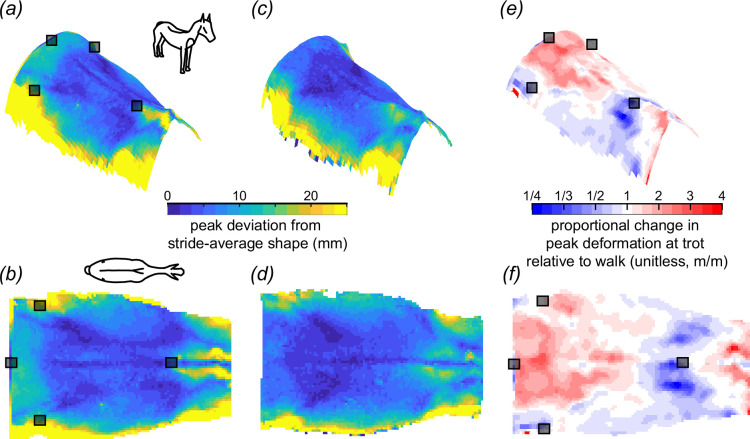
Peak deviation from stride-average shape of the equine back in trot (*a*,*b*) and walk (*c*,*d*). The deviation in walk was less than in trot, and while the deviation from the stride-average shape exhibited a qualitatively similar profile between the two, the trot exhibited greater spinal extension and cranio-caudal curvature. Views are elevated right cranio-lateral (*a*,*c*,*e*) and dorsal (*b*,*d*,*e*). Note that within the saddle-relevant region, the area of the withers (cranial thoracic spine) deformed the greatest in trot (*a*,*b*) and walk (*c*,*d*). Grey squares represent motion-capture markers placed at (in the cranio-caudal direction): T6, tuber coxae (two markers) and Cd1.

**Figure 6.**
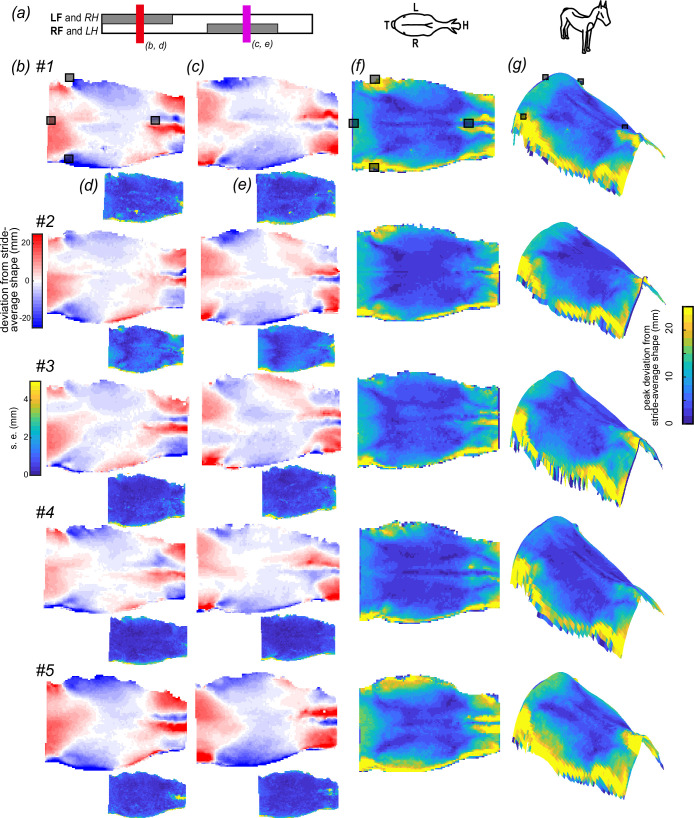
Patterns of shape change of the back in trot were consistent among horses. (*a*) We compared back shape at mid stance for each pair of limbs (red—LF and RH corresponding to *b*,*d*; purple—RF and LH corresponding to *c*,*e*). The instances are approximate mirror images of one another, reflected about the spine. (*b*,*c*) Dorsal views of the change in the back shape of five horses (#1–#5) colour-coded by the change in elevation relative to the stride-average shape at two instances in trot. (*d*,*e*) Changes in back elevation were consistent across multiple strides, as demonstrated by the low s.e.m., colour-coded across the surface. Typical errors were an order of magnitude less than the peak changes in elevation. The error presented captures errors in identifying the same instances in time, aligning or registering the shapes and measurement errors. Sample sizes for (*b*,*d*) (#1–#5) are *n* = (7,4,8,8,8), and (*c*,*e*) (#1–#5) *n* = (8,6,8,8,8). (*f*,*g*) The pattern of peak deviation of the back relative to the stride-average shape was similar across the five horses. Views are (*f*) dorsal and (*g*) right cranio-lateral. Note differences in the degree and area of the wither elevation. (*a*) LF: left forelimb; RH: right hindlimb; RF: right forelimb; LH: left hindlimb. (*a*) Inset of dorsal view of horse displaying H: head, T: tail, L: left, R: right. Grey squares represent motion-capture markers placed at (in cranio-caudal direction): T6, tuber coxae (two markers) and Cd1.

Below we discuss our reconstruction uncertainty (figure 1); the instantaneous shape of the back throughout trot and walk (figure 3); the difference between the stride-average shape and the shape of the standing horse (figure 4); the maximum change in shape of the horse’s back relative to its stride-average shape (figure 5); and, lastly, a comparison of all five horses (figure 6).

### 3.1. Estimating reconstruction error

We estimated the reconstruction error of the camera configuration and calibration by comparing the surface of a physical static horse model generated by the experimental setup to that from a high-accuracy laser scan, which served as a reference. The median absolute difference between the two reconstructions was 0.54 mm, 76.5% of points were less than 1 mm away from the scan, and 95% of points were within 1.9 mm of the laser-scanned model (figure 1*e*).

### 3.2. Change in shape of the back in trot and walk

The equine back is elevated and depressed repeatedly throughout a stride (figure 3*c,f*), with gait-specific differences (figure 3*b,e*). However, across both gaits, the regions occupied by the withers, shoulders and lateral thorax were of greatest change.

In trot, the withers and shoulder region elevated most relative to the stride-average shape of the back. Maximum elevation of the shoulder region occurred when the ipsilateral forelimb was in the mid-stance phase, with the instantaneous-average elevation of the shoulder region reaching a maximum greater than 18 mm for all horses (figure 3*b*(ii)(vi)), while the withers elevated maximally when the ipsilateral forelimb was in mid-swing phase reaching a maximum greater than 25 mm for all horses (figure 3*b*(ii)(vi)); electronic supplementary material, figures S2–S5).

Walk exhibited the opposite pattern of elevation in relation to the ipsilateral forelimb phase. Maximum elevation of the shoulder region occurred when the ipsilateral forelimb was in the early-swing phase reaching a maximum greater than 15 mm for all horses (figure 3*e*(ii)(vi)), while the withers elevated maximally when the ipsilateral forelimb was in the early-stance phase reaching a maximum greater than 9 mm for all horses (figure 3*e*(i)(v)). Interestingly, the timing of peak elevation of the withers relative to the ipsilateral forelimb was during a mid-late swing in trot and early stance in walk. Notably, the withers elevated less in walk than trot for all horses.

The elevation of the lateral thoracic region occurred when the ipsilateral forelimb was in the late-stance phase in both gaits. In all horses, the lateral thoracic region elevated more than 7 mm in trot (figure 3*b*(iii)(iv)); electronic supplementary material, figures S2–S5) and 15 mm in walk (figure 3*e*(ii)(v) and electronic supplementary material, figures S2–S5).

### 3.3. Is the shape of a standing horse the same as its stride-average shape?

The back shape of a standing horse is different from its shape during trot or walk (figure 4). The moving horse’s back was flatter and wider than the standing one, i.e. decreased craniocaudal concave-up and transverse concave-down curvatures, which manifest as depression at the withers and croup and elevation of the lateral extremes. In trot, the differences between the moving shape and standing shape deviated substantially; even if we exclude the 5% most extreme deviations, the range was still −31 mm to +35 mm (trot relative to stand). The areas of the back that differed most were: the withers, lateral thorax and, to a lesser extent, the croup. The back retained a similar pattern of elevations in trot and walk, with trot exhibiting a slightly flatter profile than walk. These conclusions were the same regardless of whether we compared the standing shape before or after exercising performed during the veterinary examination.

### 3.4. Peak deviation from the stride-average configuration in trot and walk

No region of the back remained static during trot or walk. While our previous analyses focused on the stride-average shape, here we focus on the peak deviations from that stride-average shape. If we exclude the extreme edges of the reconstruction, which exhibited greater uncertainty (figures 3 and 6), the region that moved most in trot and walk was the withers, which were elevated and depressed by ±30 mm and ±18 mm in trot and walk, respectively (trot range across horses: ±20–45 mm; walk range across horses: ±10–35 mm). The least degree of change in both gaits was along the spinal column.

The surface maps of these peak deviations were qualitatively similar between trot and walk, but the two differ in more than just scale. The peak deformation of the withers and croup was greater in trot than in walk (figure 5*e,f*). The opposite was true for the middle thoracic region, where peak deformation was greater in walk than in trot.

## 4. Discussion

We recorded the shape of the equine back in multiple horses during standing, walking and trotting using a set of four high-speed cameras and reconstructed the surface of the saddle region to quantify its patterns of gait-specific shape change and the degree of equine back mobility. While no region of the back was static, there were regions of the thoracolumbar back that moved on the order of millimetres, as opposed to centimetres, which would be good candidate regions for placing a relatively rigid saddle. We discuss the implications of these findings for saddle design by focusing on the following: (i) the changing shape of the equine back during locomotion and the anatomical reasons for these changes, (ii) differences between the average standing, walking and trotting shapes and their importance for saddle-fit assessment, (iii) the least-mobile regions of the back that are best suited for saddle-induced pressure distribution, (iv) the mobility of the withers, and (v) the importance of not positioning a saddle too far cranially on the horse. Additionally, we discuss some of the tree shape and size modifications of the traditional English saddle design and whether they are supported by our results from the point of view of equine back mobility.

### 4.1. Back shape is not fixed

The equine back changes shape throughout trot and walk (figure 3). The areas that changed elevation most are associated with soft tissue, while the areas that changed elevation least are associated with the spinal column. The mobility of the soft-tissue regions arises owing to the bulging of epaxial and extrinsic forelimb muscles, resulting from concentric contractions of the muscle bellies [24]. The increases in elevation across the back were consistent with electromyographic activity patterns of epaxial muscles, particularly the longissimus dorsi, one of the largest epaxial muscles and important for spinal stability [15]. The longissimus dorsi activates unilaterally during late stance of the ipsilateral hindlimb in both trot and walk, as well as the aerial phase in trot in dogs and horses [15,19], consistent with our elevated surface profiles (figure 3*b*(iii)(vii), *e*(i–iii)(v–vii)).

### 4.2. Back shape differs between standing and moving

The equine back during trot or walk is flatter, with a less-pronounced saddle shape than in the standing horse. Averaged over a stride, the moving horse’s withers and croup are depressed, decreasing longitudinal (cranio-caudal) concave-up curvature, while transversely the ribs are elevated decreasing transverse (medio-lateral) concave-down curvature (figure 5). Importantly, there was a greater difference between the shapes of the standing and moving horse than between the shapes of it walking and trotting.

The differences in back shape between the standing and moving horse are important to consider during saddle assessment. All saddles are fitted to the standing horse (but note: saddlers will ensure that the saddle does not substantially impede a locomoting horse’s kinematics); however, the majority of time ridden with a saddle may be spent locomoting and not standing. Selecting a tree with a longitudinal and transverse curvature to match the curved back shape of a standing horse will increase pressures applied to the back during locomotion owing to the mismatch in shape. For some horses this will be appropriate: a horse used for show or guarding may spend the majority of its ridden time standing. However, many horses are primarily ridden for regular exercise and will spend most of their time locomoting. How we best balance these different configurational demands to improve long-term well-being requires both acute analyses and long-term study. However, the expectation is that the curvature of a saddle should match the primary activity of the horse and be less pronounced for exercising horses and more pronounced for standing horses.

**Figure 7.**
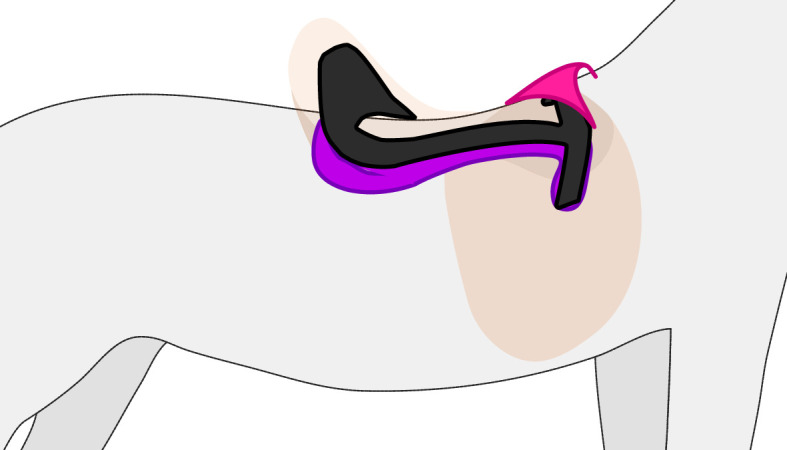
The simplified structure of an English saddle. The tree of the saddle (black) sits atop the horse (grey) providing an interface between the rider and animal. The caudal portion of the tree is supported by large pads or panels (purple), which provide cushioning and help accommodate the mismatch between the rigid tree structure and the shape of the horse. The pommel (pink) of the saddle sits above the withers. Additional saddle parts are visible as transparent tan.

Recent technological advances in three-dimensional scanning and manufacturing techniques allow for saddles to better match the standing configuration. These advances in saddle design produce more consistent fits than traditional subjective approaches [1,36,37]. We hypothesize that while scans of standing horses produce greater consistency and accurately reproduce the instantaneous shape, this is not often going to produce an objectively better saddle. As there is a mismatch in back shape between standing and moving, the saddle fit to a standing horse will not match a locomoting horse. Furthermore, the forces and pressures presented by a saddle are greatest during locomotion [38], and accounting for peak and sustained pressures must be part of the design objective. While subjective saddle-fit assessment is less accurate than technology-based methods, resulting English saddles are generally padded with centimetres of materials in the panels, which may accommodate morphing during locomotion (figure 7). Future work should assess whether three-dimensional-scan-based saddles yield better outcomes in moving horses than traditional approaches.

Scanning every horse using photogrammetry is not yet practical for saddle design. Future work on larger populations should develop a predictive model of the shape change during movement relative to standing horses, which would allow saddlers to anticipate and design around the predicted degree of back flattening relative to the horse’s current standing measurements, history and anticipated activity.

### 4.3. Saddle design to reduce back pressure

Traditional saddles interface with the horse along rigid frames known as ‘trees’ (figure 7). The rigidity of trees does not allow them to follow the changes in the shape of the back. In theory, the consequence of rigid saddles is that they apply high pressure to the mobile regions of the back, which, for poor saddle fits, can lead to muscle trauma and atrophy [39,40]. The benefit of rigid trees is that they better distribute load over the immobile regions when the fit is well aligned between tree and back.

In saddle fitting, there is no singular region that is used as the interface between tree and back. For a given horse, the area of the tree in contact with the back will depend on the type of saddle (e.g. English, western, military and racing), with the common English saddle tree being among the smaller designs, but far from the smallest. Despite varying configurations, the goal of all trees is to distribute the weight of the rider and suit the mode of riding.

To distribute pressure evenly, assuming that the soft tissues of the equine back have similar stiffness, a rigid saddle should avoid the mobile regions and should only cover the least-mobile soft-tissue regions of the back. The least-mobile areas are located in the thoracic back (figure 5), which reassuringly consists of the region covered by all types of saddle trees, but it is overall a larger region, much larger than that of the English saddle. In principle, a larger tree covering this low-mobility region would distribute pressure over a greater area of the back and better avoid detrimental focal high-pressure points.

Current saddle-fitting practice specifically guides against long saddles and suggests that the English saddle tree should not extend beyond T18 [9,36,41]. Among the concerns are that long saddles may lead to poor locomotor performance or behavioural changes owing to ‘bridging’ of the saddle between the cranial–caudal thoracic regions [11,41,42], where only the cranial and caudal regions of the tree distribute pressure. In trot and walk, our measurements of unridden horses do not suggest bridging to be a concern. Long saddles fit to a standing horse are more likely to do the opposite of bridge and instead rock craniocaudally owing to the flattening of the back during locomotion; long saddles fit to the moving horse would also not likely bridge owing to the low mobility of the region occupied by the saddle (figure 6*f,g* and electronic supplementary material, figure S1). However, bridging may be a concern in canter or gallop, or may be among the changes in back shape induced by a rider.

Interpreting studies of wider and longer saddles is difficult, as our data suggest that unless the static standing horse is the focus, then a study that increases the size of a saddle that does not fit the moving horse is really instead just further decreasing the quality of fit during movement and performing worse. Indeed our results may shed some insight into why some studies have found ‘bridged’ saddles to have lower back pain scores in ridden horses [1].

If the goal was to maximize saddle area to fit the least-mobile soft tissue of the back in our population of five horses, the saddle would extend from: just caudal to T8 to approximately 10 cm cranial to the imaginary line connecting the two tuber coxae (figure 5*b,d*). However, this area is solely based on the shape change of the equine back without a rider, and further studies will be required to assess the effect of saddle and rider on this potential saddle-tree sizing [41,43,44].

An alternative approach to reducing saddle-induced back pressure over the mobile regions is to add mechanical degrees of freedom to a saddle to accommodate the tissue movement. While additional degrees of freedom, such as hinges, will always be capable of better alignment with a moving horse (figure 5), in the same way that any mathematical-shape function is better fit with more degrees of freedom, the improvement is not necessarily substantial as the region interfacing with the tree is relatively immobile. Nevertheless, hinges could make the saddle capable of accommodating the large seasonal back shape changes, and decrease the need for regular saddle adjustments [45].

### 4.4. Patterns of withers elevation

Of the regions relevant to saddles, the areas that deformed the greatest were the withers. The withers elevated and depressed more than fourfold greater than the other areas that might interact with an English saddle. The timing of maximal withers elevation is not consistent with many obvious patterns of the ipsilateral forelimb: maximal withers elevation (i) occurred during different gait phases—mid-swing in trot and early stance in walk; (ii) does not coincide with peak ground reaction forces; and (iii) does not coincide with extreme joint angles of the scapula (figure 3) [46,47]. Across walk and trot, maximal withers elevation coincided with the late-stance phase of the contralateral forelimb. As the withers elevate in response to scapular elevation and rotation, it is likely that one or more extrinsic forelimb muscles are responsible for the scapular movement. In walking and trotting dogs, rhomboideus (a scapular elevator) activates during similar periods when the withers elevate [48].

Owing to the high mobility of the withers and the importance of this region in forelimb protraction and forward movement of the horse [24], modern saddle design and placement aim to minimize withers compression. Common saddler guidelines for avoiding withers compression position the saddle pommel elevated above the withers (figure 7) with varying amounts of suggested clearance [9,11,41]. Our study provides an experimental demonstration of the degree of withers mobility and supports the rationale of maintaining clearance of this region. We are unable to provide specific clearance guidance as maximum withers elevation varied substantially (between 5 and 25 mm), and we did not measure canter and gallop that may have even greater withers elevation. However, we can suggest that the clearance of the saddle should well exceed 25 mm with a seated rider, or alternatively, the front edge (cranial) of the saddle should be designed to sit caudal to or around the withers (figure 5), as a means of avoiding inadvertent compression of this highly mobile region.

### 4.5. The importance of the withers in saddle placement

While saddle design is important, a well-fitted saddle can still be poorly positioned by riders and result in saddle-induced injury. Our results indicate that a saddle incorrectly positioned caudally distributes its pressure on a region of low mobility. However, a saddle incorrectly positioned *cranially* encroaches on the highly mobile withers (figure 5). These results suggest that in instances of uncertain saddle placement, we would expect that an error in placing the saddle caudally, rather than cranially, would lead to less tissue impingement and produce better outcomes than the risk of impinging the withers [27]. However, shifting the saddle, and hence the rider’s weight, too far caudally will alter the kinematics of the thoracolumbar spine and the hindlimbs [38].

## 5. Conclusions

We accurately reconstructed the surface of five trotting and walking horses. Our methodology improved upon previous techniques by intentionally laterally shifting the lenses to adjust the field of view increasing spatial resolution (figure 2). We achieved millimetre-scale errors in our reconstructions of a life-sized model horse, and repeated measures of all five horses were at millimetre-scale s.e. across trot and walk (figures 1 and 3).

The shape of horses’ backs is not fixed; they morph substantially, with some regions deforming by multiple centimetres. The trot- and walk-specific patterns of shape change were repeatable within an individual and qualitatively similar between individuals (figures 3 and 6). Reassuringly, despite that there are regions of the back that deform substantially, traditional saddles sit atop soft tissues that deform by millimetres as opposed to centimetres. Therefore, a saddle fit to a walking or trotting horse can be a rigid structure and would be suitable for distributing load to the tissue below.

Problematically, the shape of the standing horse was substantially different from that of either the walking or trotting horse; the stride-average shape of the horse in walk and trot was more similar. Standard saddle-fitting practice fits the saddles to the standing horse and verifies that it is acceptable by watching the horse move. Our results provide some clarity to the importance of this second step by demonstrating how substantially different the shape of the moving horse is from its standing configuration. For many sport horses, the majority of the time ridden is spent locomoting, and the better practice, albeit not yet viable, might be fitting the saddle to the moving horse and verifying that it is acceptable when standing.

Amongst the most mobile regions of the back were the withers. This critical region is generally more likely to be impinged by saddles, and our results highlight why increased care is necessary for designing and fitting around the withers. As the withers are adjacent to the saddle, we predict that errors in saddle positioning cranially and caudally are not equal: that is, small errors in saddle positioning caudally are less likely to impinge mobile tissue than equal errors cranially.

Finally, our results provide insight into the mobility of the back in an unridden horse. The exciting next steps are to identify how these results change with the load and dynamics of a rider, and how we might best design saddles around the deforming loaded back of horses to improve their welfare.

## Data Availability

All data are available within the figures and electronic supplementary material [49].
